# Changes in HIV-Related Cervical Cancer Over a Decade in Côte d'Ivoire

**DOI:** 10.1200/GO.21.00006

**Published:** 2021-05-27

**Authors:** Antoine Jaquet, Simon Boni, Boris Tchounga, Kouassi Comoe, Aristophane Tanon, Apollinaire Horo, Isidore Diomandé, Judith Didi-Kouko Coulibaly, Didier K. Ekouevi, Innocent Adoubi

**Affiliations:** ^1^ University of Bordeaux, Inserm, French National Research Institute for Sustainable Development (IRD), Bordeaux, France; ^2^ Programme National de Lutte contre le Cancer (PNLCa), Abidjan, Cote d'Ivoire; ^3^ Programme PACCI /site ANRS Abidjan, Abidjan, Cote d'Ivoire; ^4^ Elizabeth Glaser Pediatric AIDS Foundation, Yaoundé, Cameroon; ^5^ Service de cancérologie, Centre Hospitalier Universitaire de Treichville, Abidjan, Côte d'Ivoire; ^6^ Service des Maladies Infectieuses et Tropicales (SMIT), CHU de Treichville, Abidjan, Côte d'Ivoire; ^7^ Service de Gynécologie obstétrique, CHU de Yopougon, Université Félix Houphouet Boigny, Abidjan, Côte d'Ivoire; ^8^ Service anatomopathologie, Centre Hospitalier Universitaire (CHU) Cocody, Abidjan, Cote d'Ivoire; ^9^ Centre National de Radiothérapie et d'Oncologie Médicale Alassane Ouattara, Abidjan, Cote d'Ivoire; ^10^ Université de Lomé, Département de Santé Publique, Lomé, Togo

## Abstract

Major improvements have occurred in access to invasive cervical cancer (ICC) screening in HIV-infected women over the past decade in sub-Saharan Africa. However, there is limited information on changes in the burden of HIV-related ICC at a population level. Our objective was to compare HIV-related ICC over a decade and document factors associated with HIV infection in women with ICC in Côte d'Ivoire.

**METHODS:**

A repeated cross-sectional study was conducted in referral hospitals of Abidjan, Côte d'Ivoire, through the 2009-2011 and 2018-2020 periods. Women diagnosed with ICC were systematically tested for HIV. Demographics, ICC risk factors, cancer stage (International Federation of Gynecology and Obstetrics), and HIV characteristics were collected through questionnaires. Characteristics of HIV-related ICC were compared between the periods, and factors associated with HIV in women diagnosed with ICC in 2018-2020 were documented through a multivariable logistic model.

**RESULTS:**

During the 2009-2011 and 2018-2020 periods, 147 and 297 women with ICC were diagnosed with estimated HIV prevalence of 24.5% and 21.9% (*P* = .53), respectively. In HIV-infected women, access to antiretroviral treatment increased from 2.8% to 73.8% (*P* < 10^−4^) and median CD4 cell count from 285 (IQR, 250-441) to 492 (IQR, 377-833) cells/mm^3^ (*P* = .03). In women diagnosed with ICC during the 2018-2020 period, HIV infection was associated with a less advanced clinical stage (International Federation of Gynecology and Obstetrics I or II stage) (adjusted OR, 2.2 [95% CI, 1.1 to 4.4]) and with ICC diagnosis through a systematic screening (adjusted OR, 10.5 [95% CI, 2.5 to 45.5]).

**CONCLUSION:**

Despite a persistently high proportion of HIV-related ICC over time in Côte d'Ivoire, HIV was associated with less advanced clinical stage at ICC diagnosis. Recent improvements in ICC screening services across HIV clinics might explain this association and support their implementation across non-HIV health facilities.

## BACKGROUND

Despite major achievements for its prevention, invasive cervical cancer (ICC) incidence is still on the rise and remains the second cause of cancer as well as the leading cause of death-related cancer among women living in sub-Saharan Africa (SSA). In this context, Côte d'Ivoire is particularly affected with an age-standardized incidence and mortality rate from ICC of 31.2 cases and 22.8 deaths per 10^5^ women in 2018.^1,2^ Infection with HIV is known to increase the risk of ICC and has been classified as an AIDS-defining disease since the early HIV epidemic. In SSA, women diagnosed with ICC are over four times more likely to be HIV-infected compared to women with no ICC.^3,4^ Major improvements have occurred in access to HIV care during the past decade with the implementation of universal antiretroviral therapy (ART).^5^ Although ART initiation has shown to reduce the incidence of some AIDS-related malignancies such as Kaposi sarcoma in HIV-infected people living in SSA, its impact on the risk of ICC remains unclear.^6-8^ However, a recent meta-analysis suggested a protective effect of ART on the risk of cervical precancerous lesions and ICC.^9^ As ART continues to expand in SSA, more evidences are needed to characterize its potential impact on the subsequent risk of AIDS-defining malignancy such as ICC in HIV-infected women.

**CONTEXT**

**Key Objective**
To compare characteristics of HIV-related cervical cancer diagnosed in the urban area of Abidjan over a decade. Although HIV constitutes a major risk factor for cervical cancer, limited information is available toward the evolution of HIV-related cervical cancer over time in sub-Saharan Africa in a context of major progress in HIV care and cervical cancer screening programs.
**Knowledge Generated**
The prevalence of HIV infection in women diagnosed with cervical cancer in Abidjan remained persistently high (> 20%) compared with the 2.4% HIV prevalence reported in the adult general population in 2020. HIV infection was associated with less advanced clinical stage at diagnosis and higher access to cancer diagnosis through systematic screening.
**Relevance**
Tumor downstaging associated with HIV infection might reflect efforts to scale up cervical cancer screening in HIV clinics over time. Systematic screening should be pursued and expanded to women regardless of their HIV status in Côte d'Ivoire.

During the past decade, a growing number of ICC screening programs have been piloted and/or implemented in SSA, many of them partly or fully conducted through integrated HIV care services.^10,11^ In Côte d'Ivoire, pilot programs initially targeting HIV-infected women have been progressively extended to health care facilities with no HIV care program.^12-14^ However, there is limited information on how improved access to HIV prevention and care services combined with the increased access to ICC screening may have influenced the characteristics of HIV-related ICC. Our objective was to compare the characteristics of HIV-related ICC over a decade and document factors associated with HIV infection in women diagnosed with ICC during the 2018-2020 period in Côte d'Ivoire.

## METHODS

### Population and Design

A repeated cross-sectional study was conducted in Abidjan, the economic capital of Côte d'Ivoire, during the 2009-2011 and 2018-2020 periods. During these two 24-month periods (May 2009-June 2011 and July 2018-June 2020), clinical units located in the urban area of Abidjan, known to manage women with gynecologic malignancies were asked to systematically include all adult women (≥ 18 years old) attending with a suspected or confirmed diagnosis of ICC. Cervical biopsies and histologic examination by the local pathology unit were systematically proposed and financially supported by the research project, when appropriate. During the 2009-2011 period, four units from the three public referral hospitals of Abidjan (three gynecologic units and one cancer unit) were involved as previously reported in a first large case-referent study on cancer and HIV conducted in West Africa.^3^ The 2018-2020 period covered all units potentially managing ICC from the public sector (including the four previously mentioned units plus the recently opened radiotherapy center) as well as the three major clinics from the private sector with the capacity to manage ICC in the urban area of Abidjan.

### Collected Information

Women enrolled during the two periods were administered a similar structured questionnaire to collect sociodemographic characteristics including age, formal education (categorized as no school, primary school, and secondary school and over), personal monthly income, age at first sexual intercourse, parity, tobacco use (categorized as current or former tobacco users *v* never users), and current hormonal contraceptive use. During both study periods, the questionnaire was administered by clinical monitors specifically trained for this purpose. Cancer clinical stage at ICC diagnosis was assessed based on the International Federation of Gynecology and Obstetrics (FIGO) staging system.^15^ Based on available information after the initial assessment of the tumor extension, clinical stage at diagnosis was reported by clinicians and dichotomized as early (stage I and II) or advanced (stage III and IV) disease.

Additional information was collected during the 2018-2020 period including the existence of any personal health insurance coverage. Prediagnosis history was also documented including date of first reported ICC-related symptoms, date of first consultation at an ICC referral center, whether ICC was diagnosed following attendance to a systematic ICC screening without prior symptoms or not, and attendance to a traditional healer or using any traditional treatment for ICC-related symptoms before diagnosis.

During these two periods, a nationally approved rapid HIV test (Determine, Abbott Diagnostics) was systematically performed by collecting capillary blood by a finger prick test at the time of interview.^16^ In case of positive result, a venous blood sample was collected for confirmation purposes, according to the national algorithm of Côte d'Ivoire. Participants with a previously known HIV infection were surveyed with regards to their HIV characteristics including their date of first HIV diagnosis, ART use, last known CD4 count (and last known HIV viral load measure, only for the 2018-2020 period). These HIV-related data were collected combining participants' interview with specific data request to HIV programs following participants with a previously documented HIV infection.

The present research has been performed in accordance with the Declaration of Helsinki and has been approved by the national ethics committee of Côte d'Ivoire [no 011-19/MSHP/CNESVS-kp]. All women enrolled in 2009-2011 and 2018-2020 provided their informed and written consent before participate.

### Statistical Analysis

Participant characteristics were compared according to the presence or absence of HIV infection and between studied periods using Pearson's χ^2^ test or Fisher's exact test when appropriate for categorical variables. Given the existence of some non-normally distributed continuous variables, central tendencies were reported through median values with their IQR and compared with Mann-Whitney test or Kruskal-Wallis tests when appropriate. An unconditional logistic regression model was used to estimate the association between HIV infection and participant characteristics in women diagnosed with ICC during the 2018-2020 period. Odds ratio estimates were reported with their 95% CI. A multivariable model was computed following a stepwise descending procedure. Available factors associated with a *P* value < .2 were systematically included in a full model. Additional relevant potential confounders known to be associated with HIV or ICC such as tobacco use, oral contraceptive use, or socioeconomic status (education and income) were also considered regardless of their statistical association and included in the initial multivariate model. Modification effects and potential interaction between available confounders, clinical staging, and HIV infection were systematically assessed. The goodness of fit of the model was then assessed using the Akaike information criterion (AIC), with a lower value of the AIC suggesting a better prediction of the model. Confounders that were not significantly associated with HIV infection and did not add any significant prediction to the model based on the AIC were sequentially removed. All statistical analyses were performed using SAS software, version 9.4 (SAS Institute Inc, Cary, NC).

## RESULTS

### Changes in HIV-Related ICC Between the 2009-2011 and the 2018-2020 Periods

During the 2009-2011 and 2018-2020 periods, 147 and 297 women with ICC were included, with a median (IQR) age at ICC diagnosis of 49 (IQR, 40-57) years and 51 (IQR, 43-60) years (*P* = .01), respectively. Diagnosis of ICC was histologically confirmed in all cases included during the 2018-2020 period while 128/147 (87.0%) had histologic confirmation during the 2009-2011 period. Compared to women diagnosed with ICC in 2009-2011, those diagnosed in 2018-2020 presented with higher tobacco use (13.2% *v* 2.7%; *P* < .001) and higher hormonal contraceptive use (13.1% *v* 0.7%; *P* < .0001). An advanced FIGO clinical stage (III and IV) was reported in 74.5% and 76.2% of participants during the 2009-2011 and the 2018-2020 periods, respectively (*P* = .72).

The estimated proportion of women with ICC who were HIV-infected during these two periods was 24.5% and 21.9% (*P* = .53), respectively. The median age of HIV-infected women at ICC diagnosis was 44 (IQR, 36-48) years in 2009-2011 versus 46 (IQR, 41-51) years in 2018-2020 (*P* = .14). Although no significant association in advanced clinical stage was reported according to HIV status during the 2009-2011 period, HIV-infected women were less likely to present with an advanced clinical stage compared with their HIV-uninfected counterparts during the 2018-2020 period (61.3% *v* 80.5%; *P* < .01) (Table 1). During the 2009-2011 period, 61.1% of HIV-infected women with ICC were unaware of their HIV status before study participation versus only 13.8% of HIV-infected women with ICC in 2018-2020. Concomitantly, access to ART increased from 2.8% to 73.8% between the two periods (*P* < .0001) (Table 2). In women with a previously documented HIV infection, median CD4 cell count measures significantly increased over time from 285 (IQR, 250-441) cells/mm^3^ in the 2009-2011 period to 492 (IQR, 377-833) cells/mm^3^ in the 2018-2020 period (*P* = .03). Among HIV-infected women on ART at their ICC diagnosis during the 2018-2020 period, median time since ART initiation was 3.8 years (IQR, 1.2-8.3 years).

**TABLE 1:**
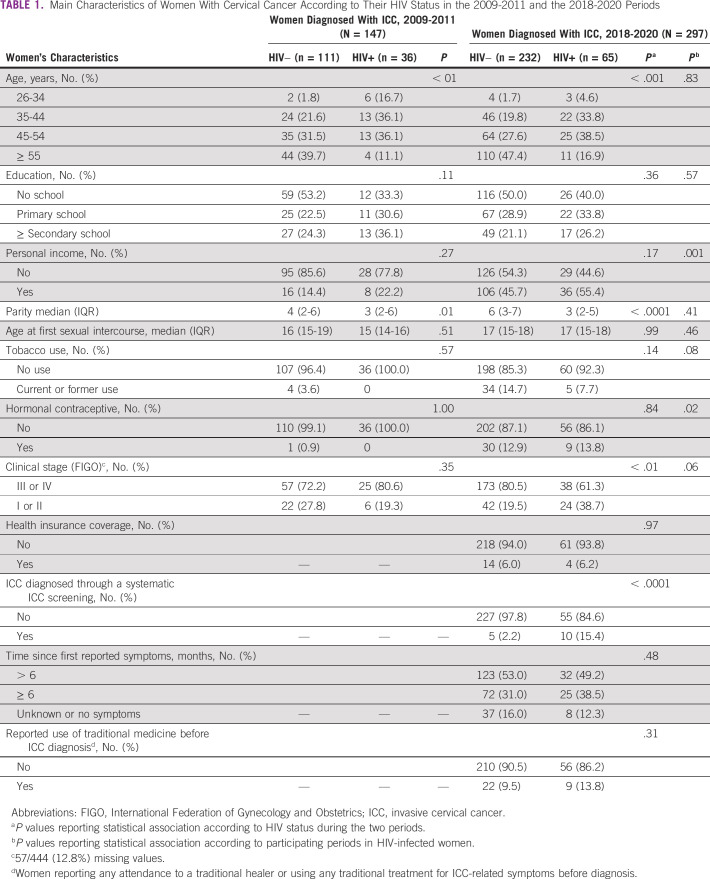
Main Characteristics of Women With Cervical Cancer According to Their HIV Status in the 2009-2011 and the 2018-2020 Periods

**TABLE 2:**
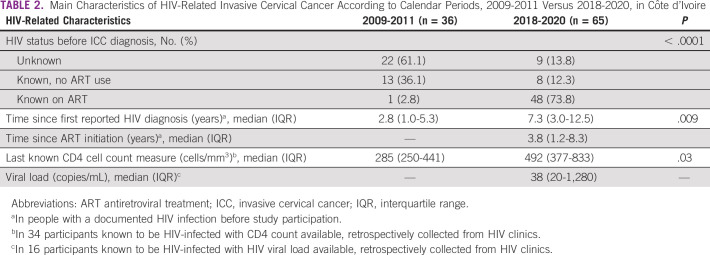
Main Characteristics of HIV-Related Invasive Cervical Cancer According to Calendar Periods, 2009-2011 Versus 2018-2020, in Côte d'Ivoire

### Factors Associated With HIV Infection in Women Diagnosed with ICC During the 2018-2020 Period

Of the 297 women with ICC included during the 2018-2020 period, ICC diagnosis was subsequent to a systematic screening with no pre-existing symptoms in 15 (5.0%) of women. Women living with HIV were more likely to be diagnosed through systematic screening (15.4%) compared with HIV-uninfected women (2.2%) (*P* < .0001). For symptomatic women, the median time between first reported ICC-related symptoms and first consultation at a cancer referral center was 3.9 (IQR, 1.4-9.2) months with no significant difference according to HIV status (*P* = .29). Access to traditional medicine before ICC diagnosis was reported by 13.8% of HIV-infected women and 9.5% of HIV-infected women (*P* = .31). An existing health insurance coverage was reported by 18 (6.1%) women with no differences according to HIV status (*P* = .97). In a multivariable analysis, HIV infection was significantly associated with a less advanced clinical stage (FIGO I or II stage) at ICC diagnosis (adjusted OR [aOR], 2.2 [95% CI, 1.1 to 4.4]), ICC diagnosed through a systematic screening [aOR, 10.5 (95% CI, 2.5 to 45.5)], and inversely associated with age ≤ 55 years (aOR, 0.2 [95% CI, 0.1 to 0.7]) (reference group 26-34 years of age) (Table 3).

**TABLE 3:**
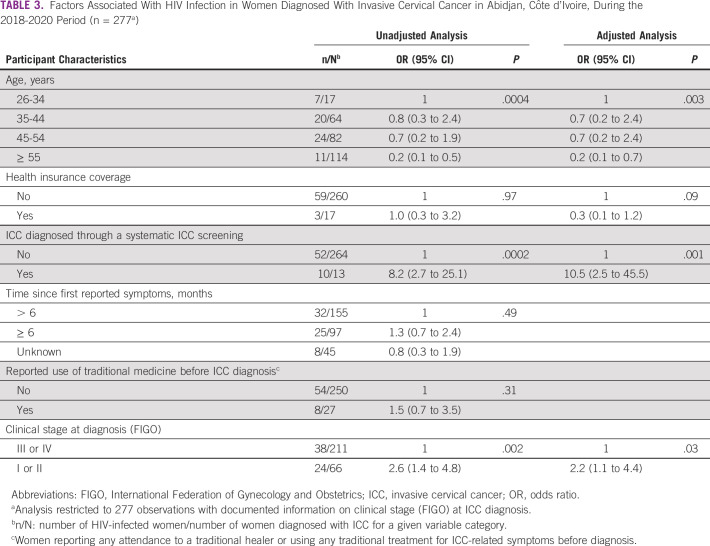
Factors Associated With HIV Infection in Women Diagnosed With Invasive Cervical Cancer in Abidjan, Côte d'Ivoire, During the 2018-2020 Period (n = 277^a^)

## DISCUSSION

Major improvement in access to HIV care was observed in women diagnosed with ICC in Côte d'Ivoire through the past decade, in line with reports from the general population (UNAIDS 2020 report).^17^ Thanks to these achievements in the fight against HIV/AIDS, the prevalence of HIV infection in the adult general population has decreased from 3.7% (95% CI, 3.2 to 4.2) to 2.4% (95% CI, 2.0 to 2.8) over the past decade in Côte d'Ivoire.^17^ Unlike 10 years ago, the majority of HIV-infected women diagnosed with ICC in 2018-2020 where aware of their HIV status and currently on ART. Women diagnosed with ICC in the 2018-2020 harbored significantly higher CD4 count measures compared with women diagnosed through the 2009-2011 period. However, despite these major improvements in access to care and ART use among HIV-infected women, the proportion of women diagnosed with ICC and infected with HIV remained high and stable over time. A previous meta-analysis has suggested that access to ART and immune restoration have a protective effect on the occurrence of ICC in HIV-infected women.^9^ Although our study was not designed to assess the impact of access to ART on the occurrence of ICC, a protective effect of ART should ultimately translate into a decrease in the attributable fraction of HIV in ICC and, therefore, in a decrease of the subsequent proportion of HIV-related ICC. In Southern Africa, a cohort analysis documenting the incidence of ICC among 10,640 HIV-infected women followed for a median time after ART initiation of 2.1 years (IQR, 0.7-4.1 years) did not observe any decline in ICC incidence rates by time since ART initiation.^18^ Although ART might confer a certain amount of prevention against ICC, growing evidence suggest that this will not translate into a major decrease in the burden of ICC in HIV-infected women before many years. It is, therefore, essential to increase the support of ICC screening programs integrated in HIV clinics as these women remained particularly at risk despite ART use and immune restauration.

Tobacco and hormonal contraceptive use increased over time regardless of HIV status in Côte d'Ivoire. Exposure to smoked or chewed tobacco as well as prolonged exposure to hormonal contraceptive use has shown to increase the risk of premalignant cervical lesions and ICC.^19,20^ Women in Côte d'Ivoire, as in many resource-constraint settings, are increasingly confronted to a double burden of traditional ICC risk factors including high exposure to oncogenic human papillomaviruses combined with a growing exposure to Western lifestyle risk factors such as tobacco use. Prevention programs implementing ICC screening through health care facilities such as HIV clinics should be aware of these changes. This could be particularly relevant for tobacco use, which has shown to be higher in HIV-infected people even in low-income countries.^21^ Preventive approaches against tobacco use could be considered in combination with ICC screening through prevention messages and targeted smoking cessation programs for active tobacco users.

During the 2018-2020 period, and unlike the 2009-2011 period, HIV-infected women diagnosed with ICC were less likely to present with an advanced clinical stage compared with their HIV-uninfected counterparts. In addition, during the 2018-2020 period, HIV-infected women were more likely to access ICC diagnosis through a systematic screening. These findings suggest an improved access to ICC preventive and care services for HIV-infected women. Indeed, HIV-infected women diagnosed with ICC are now mainly known to be HIV-infected and regularly followed up for their HIV disease providing more opportunities in their access to care for other conditions including malignancies. In Côte d'Ivoire, ICC screening programs have been initially implemented in HIV clinics before being extended to other health care facilities. These arguments might support the hypothesis of an enhanced access to ICC care among HIV-infected women. However, the results from previous studies on the association between ICC clinical stage and HIV status are conflicting. A previous study conducted in women diagnosed with ICC during the 2008-2012 period in a referral hospital in Ethiopia reported an almost 1.5 times increased risk of diagnosis at a more advanced stage in HIV-infected women compared with HIV-negative women.^22^ Alternatively, Menon et al^23^ reported a similar association between early clinical stage and HIV infection in 315 women diagnosed with ICC between 2003 and 2010 in Uganda. Both studies were conducted in women diagnosed with ICC many years ago, when HIV care and ICC screening were clearly less available than nowadays. It is therefore important to provide more recent estimates of this association between HIV infection and ICC stage from other settings in SSA.

The cross-sectional nature of the study prevents from drawing any inferential relationship between HIV infection and its impact on the incidence of ICC over time. Indeed, the impact of HIV infection on the burden of cancers usually relies on cohort study design and record linkage studies with data extracted from population-based cancer registries. However, in most resource-limited settings, challenges associated with the documentation and continuous recording of cancers over time prevent from conducting these longitudinal approaches. Alternatively, the replication of cross-sectional studies over time using similar methods in the same catchment area enables the documentation of potential evolution in cancer characteristics providing informative and useful data to clinicians and decision makers. Because of limited available data at the referral center level to perform a precise FIGO staging, our definition of an advanced stage at diagnosed did not followed the standard definition used for eligibility to a curative surgery (stage I, IIa *v* stage IIb, III, or IV). Therefore, the reported difference in the proportion of advanced clinical stage might not reflect a difference in access to curative treatment and ultimately enhanced survival.

Our study population does not reflect the true number of ICC that occurred in Abidjan or Côte d'Ivoire during these two periods. Indeed, an unknown proportion of women suffering from ICC never accessed any health care facilities and those who access primary care facilities never went to referral centers. The increase in the number of women with ICC between the two periods is partly related to the participation of private clinics during the 2018-2020 period. However, only 51 women were recruited through the private sector accounting for 17% of the overall number of ICC included during the 2018-2020 period. Although the catchment area of the participating hospitals remained quite similar over time, the overall population living in the urban area of Abidjan increased from 4.9 to 6.3 million of habitants between the two periods, potentially contributing to a higher absolute number of ICC in the latter period.^24^ However, the urban area of Abidjan remains the only location providing treatment for ICC in the country as well as the great majority of pathology units able to diagnose ICC. Although a few diagnoses might be reported outside this catchment area, women diagnosed with ICC should be ultimately referred to one of these referral centers.

In conclusion, characteristics of HIV-related ICC have significantly evolved over the past 10 years with now most HIV-infected women already on care and presenting with less advanced HIV disease. These achievements toward HIV care did not translate into a reduced HIV prevalence in women referred to care for ICC between 2018 and 2020 in Côte d'Ivoire. However, HIV-infected women presented with a lower proportion of advanced ICC. This finding supports the need to continue and expand ICC screening services into pre-existing health care facilities such as HIV clinics or family planning centers. Whether this greater access to early ICC diagnosis translate into better survival in HIV-infected women remains to be determined.

## DATA SHARING STATEMENT

Complete data for this study cannot be posted in a supplemental file or a public repository because of confidentiality issues. However, the dataset analyzed during the current study are available from the corresponding author antoine.jaquet@u-bordeaux.fr on reasonable request.
